# Pulmonary artery reconstruction using autologous pulmonary vein for surgical treatment of locally advanced lung cancer: a case report

**DOI:** 10.1186/s40792-016-0174-1

**Published:** 2016-05-24

**Authors:** Ayako Hirai, Shuichi Shinohara, Taiji Kuwata, Masaru Takenaka, Yasuhiro Chikaishi, Soichi Oka, Koji Kuroda, Naoko Imanishi, Fumihiro Tanaka

**Affiliations:** Department of Thoracic Surgery, University of Occupational and Environmental Health, 1-1 Iseigaoka, Yahatanishi-ku, Kitakyusyu-shi, Fukuoka, 807-8555 Japan

**Keywords:** Lung cancer, Pulmonary artery reconstruction, Pulmonary vein conduit

## Abstract

**Background:**

Resection and reconstruction of the pulmonary artery during lobectomy is a safe and effective procedure for centrally located lung cancer. We usually choose a pericardial conduit to repair a large defect of the pulmonary artery. The use of an autologous pulmonary vein conduit for reconstruction was first described in 2009.

**Case presentation:**

A 64-year-old woman with left upper lung adenocarcinoma with mediastinal and hilar adenopathy was referred to our hospital. Hilar nodes had extensively infiltrated the pulmonary artery. We interposed an autologous superior pulmonary vein between the cut ends of the pulmonary artery. She was discharged without any complication on the ninth postoperative day.

**Conclusions:**

A pulmonary vein conduit is a good option for reconstruction of the pulmonary artery. We report the successful use of an autologous pulmonary vein conduit.

## Background

According to annual surveys performed by the Japanese Association for Thoracic Surgery in 2013, pulmonary artery (PA) reconstruction for surgical treatment of primary lung cancer was performed in 227 of 37,008 cases (0.61 %) [1]. Despite the small number of cases, it is important to acquire techniques for preservation of pulmonary function and avoidance of pneumonectomy. The ideal prosthetic material for a conduit interposed within the PA is debated. We describe successful PA reconstruction with autologous pulmonary vein (PV) in the surgical treatment of left lung cancer.

## Case presentation

**Fig. 1:**
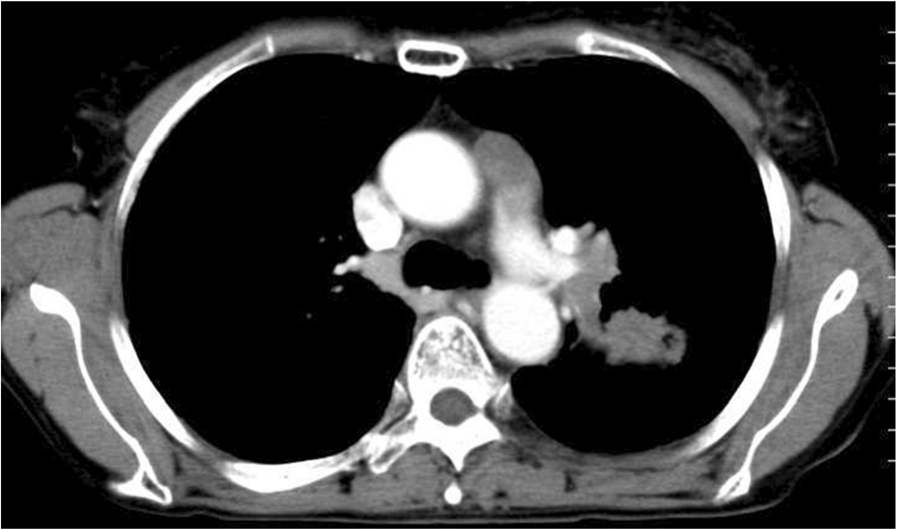
Preoperative computed tomography. Hilar lymph nodes infiltrating the pulmonary artery, and mediastinal lymph node (#6) and main tumor of S1 + 2 segments

**Fig. 2:**
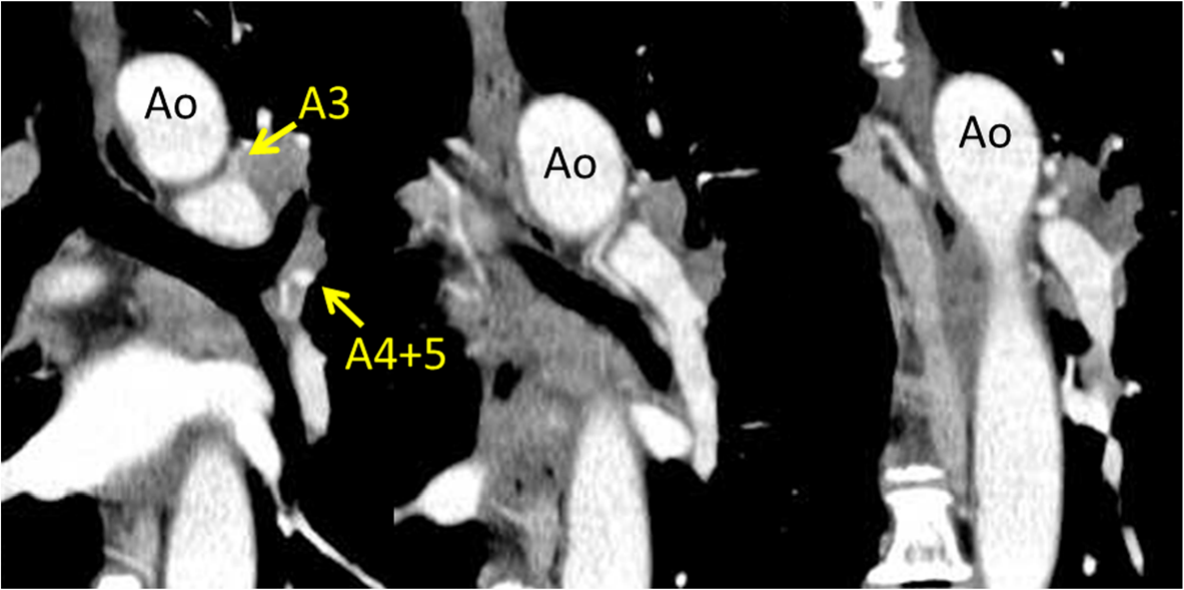
Preoperative computed tomography (serial section of coronal planes). Hilar lymph nodes infiltrating the PA beginning at the first branch to the lingular artery

A 64-year-old woman with a 5.5-cm tumor in the left upper lobe and lymphadenopathy of #6 and #12u was referred to our department (Figs. 1 and 2). She was diagnosed with cT2b N2 M0 adenocarcinoma and underwent induction therapy with carboplatin and paclitaxel weekly for 5 weeks and concurrent radiotherapy of 50 Gy in five weekly fractions. The tumor was reduced in size by 35 % by Response Evaluation Criteria In Solid Tumors (RECIST) criteria, and surgical resection was planned.

**Fig. 3:**
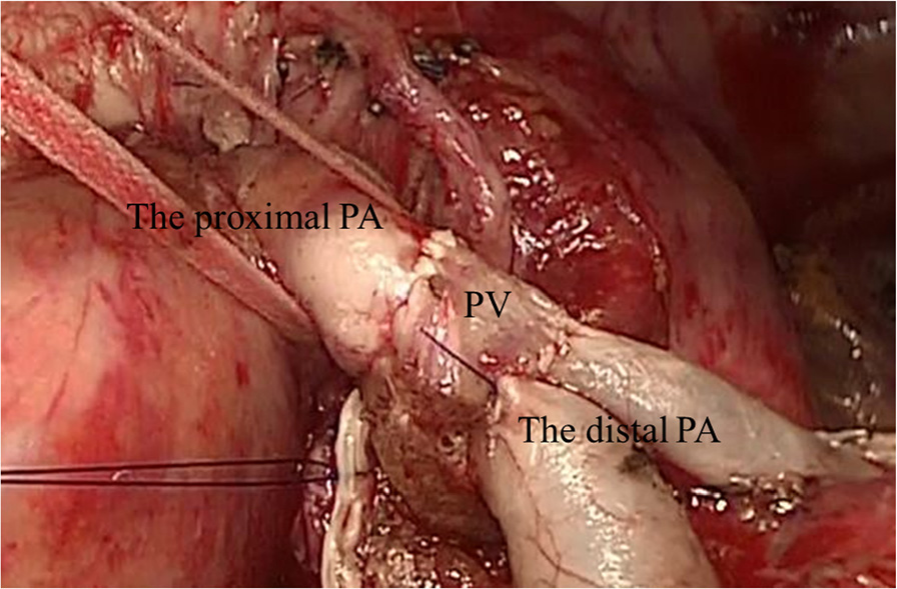
Intraoperative view after reconstruction of left PA. The PV conduit lies between the proximal (left main PA) and distal (A6, A. basalis) PA. PA, pulmonary artery; PV, pulmonary vein

A left posterolateral thoracotomy was performed. The tumor was located in the S1 + 2 segment and had invaded the interlobar pleura to involve the S6 segment. There was extensive hilar lymph node infiltration of the PA beginning at the first branch to the lingular artery. Because there was no invasion of the bronchus, we felt that conduit interposition between the PA would avoid anastomotic tension. We opened the pericardial sac and isolated the root of the main PA. The PA was clamped proximally and distally, without intravenous heparin administration, and resected. Actually, we tried to isolate the PA after the PA was clamped, but the hilar lymph node was not separated. While left upper lobectomy and wedge resection of the lower lobe were being performed, the superior PV was sutured intrapericardially, using a linear stapler, as close as possible to the left atrium. The PV was cut distally intrapericardially, and the length of PV microscopically free of cancer was 1.5 cm. After dissection of the pulmonary ligament, the PV was of sufficient length to compensate for the PA defect. We interposed the autologous PV between the proximal (left main PA) and distal (A6, A. basalis) PA and performed end-to-end anastomoses with 5-0 monofilament non-absorbable suture material (Fig. 3). The bronchial stump and PA anastomoses were separated with an intercostal muscle flap. Operative duration was 4 h 12 min and blood loss was 490 mL.

Histologic examination of the tumor revealed ypT2a N1 M0 invasive adenocarcinoma. The patient was discharged on the ninth postoperative day without complication and has remained healthy. Brain metastases appeared 18 months after the operation, but there was no local recurrence. The patient has been treated with crizotinib for 6 months and has had no progression.

## Discussion

PA sleeve resection was first reported in 1967 by Gundersen [2], and thus far the use of various materials to perform PA reconstruction with patch repair or conduit interposition has been reported. Conduit interposition is the best choice for cases in which there is an extensive PA defect. Artificial materials, such as polytetrafluoroethylene, carry a high risk of thrombosis and require long-term anticoagulation. Rendina et al. [3] described the use of an autologous pericardial conduit, which is widely used. Autologous pericardium is available on both sides of the chest and can provide sufficient tissue for repair of a large defect. However, its tendency to shrink and curl makes adaptation and suturing to the vascular wall more difficult. Cerezo et al. [4] reported the first use of an autologous PV conduit in 2009, and in a report of one case of autologous PV conduit and seven cases of patch repair, Puma et al. [5] described successful PA reconstruction with an autologous PV conduit that could be performed safely and without recurrence.

In our case, there was extensive invasion of hilar lymph nodes into the PA, but sleeve bronchial resection was not necessary. Fortunately, the tumor was located in the left chest and did not involve the superior PV intrapericardially. We could have chosen a pericardial conduit, but in this case, the autologous PV conduit was naturally tubular and of sufficient length to repair the PA defect. The length of the resected PV has been reported to be from 15 to 30 mm [3, 5, 6], but a sufficiently long PV is not always possible. The most suitable material should be determined for each case. The use of autologous PV is also an oncological problem because of the persistence of microscopic of cancer cells, and long-term results are not clear. In 2014, D’Andrilli et al. [6] reported medium-term results of nine cases of PA reconstruction with an autologous PV conduit. Tumor recurrence was observed in two patients (one local, one systemic) but no evidence of recurrence was found at the site of vascular reconstruction. They demonstrated this technique to be a feasible and effective option with acceptable medium-term results. In the present case, although distant metastases did develop, no recurrence was found at the anastomotic site.

## Conclusions

PA reconstruction is a good procedure for parenchyma-sparing resection. Although the use of an autologous PV is rarely required, under certain conditions, it can be a good option for PA reconstruction.

## Abbreviations

PA, pulmonary artery; PV, pulmonary vein; RECIST, response evaluation criteria in solid tumors
